# Cytotoxic T-Lymphocyte Antigen-4 in Colorectal Cancer: Another Therapeutic Side of Capecitabine

**DOI:** 10.3390/cancers13102414

**Published:** 2021-05-17

**Authors:** Afshin Derakhshani, Shahryar Hashemzadeh, Zahra Asadzadeh, Mahdi Abdoli Shadbad, Farnaz Rasibonab, Hossein Safarpour, Vahid Jafarlou, Antonio Giovanni Solimando, Vito Racanelli, Pankaj Kumar Singh, Souzan Najafi, Darya Javadrashid, Oronzo Brunetti, Nicola Silvestris, Behzad Baradaran

**Affiliations:** 1 Immunology Research Center, Tabriz University of Medical Sciences, 5166/15731 Tabriz, Iran; derakhshania@tbzmed.ac.ir (A.D.); asadzadehz@tbzmed.ac.ir (Z.A.); abdolim@tbzmed.ac.ir (M.A.S.); rasibonabf@tbzmed.ac.ir (F.R.); najafis@tbzmed.ac.ir (S.N.); javadrashidd@tbzmed.ac.ir (D.J.); 2 IRCCS Istituto Tumori “Giovanni Paolo II” of Bari, 70124 Bari, Italy; dr.oronzo.brunetti@tiscali.it; 3 Tuberculosis and Lung Disease Research Center, Tabriz University of Medical Sciences, 5166/15731 Tabriz, Iran; Hashemzadehs@tbzmed.ac.ir; 4 Department of General & Vascular Surgery, Imam Reza Hospital, Tabriz University of Medical Sciences, 5166/15731 Tabriz, Iran; jafarlouv@tbzmed.ac.ir; 5 Student Research Committee, Tabriz University of Medical Sciences, 5166/15731 Tabriz, Iran; 6 Cellular & Molecular Research Center, Birjand University of Medical Sciences, 9717853577 Birjand, Iran; h.safarpour@bums.ac.ir; 7 Department of Biomedical Sciences and Human Oncology, School of Medicine, Aldo Moro University of Bari, 70124 Bari, Italy; antonio.solimando@uniba.it (A.G.S.); vito.racanelli1@uniba.it (V.R.); 8 Department of Radiation Oncology Mayo Clinic, 4500 San Pablo Rd S, Jacksonville, FL 32224, USA; Singh.pankaj@mayo.edu; 9 Department of Immunology, Faculty of Medicine, Tabriz University of Medical Sciences, 5166/15731 Tabriz, Iran

**Keywords:** CTLA-4, capecitabine, chemotherapy, immunotherapy, immune checkpoint inhibitors, colorectal cancer, immune checkpoint

## Abstract

**Simple Summary:**

Colorectal cancer (CRC) begins when normal cells turn out of balance, and a tumor is formed in the lining of the colon or rectum. Cytotoxic T-lymphocyte protein 4 (CTLA-4) is a potent molecule that could inhibit T cell activation. Here, we analyzed this molecule in the tissue samples and cell lines of colorectal cancer to reveal the mechanism of this inhibitory molecule in CRC. Our result showed an increasing trend of CTLA-4 in tissues and cell lines. Finally, capecitabine as an approved drug in CRC could suppress this inhibitory molecule. It can be concluded that the inhibition of this inhibitory molecule can re-active the immune cells, especially T cells, in CRC patients, which boosts the immune cells against the tumor.

**Abstract:**

Cytotoxic T lymphocyte antigen-4 (CTLA-4) is an inhibitory immune checkpoint that can be expressed in tumor-infiltrating lymphocytes and colorectal cancer (CRC) cells. This immune checkpoint can attenuate anti-tumoral immune responses and facilitate tumor growth and metastasis. Although capecitabine is an effective chemotherapeutic agent for treating CRC, its effect on the tumoral CTLA-4 expression remains unclear. In the current research, we applied the GSE110224 and GSE25070 datasets to characterize CTLA-4 expression in CRC patients. Then, we analyzed CTLA-4 expression in CRC samples, HT-29, HCT-166, and SW480 cell lines using real-time PCR. Our bioinformatic results have highlighted the overexpression of CTLA-4 in the CRC tissues compared to the adjacent non-tumoral tissues. Our in vitro studies have indicated that SW480 cells can substantially overexpress CTLA-4 compared to HT-29 and HCT 116 cells. In addition, capecitabine can remarkably downregulate the expression of CTLA-4 in SW480 cells. Collectively, capecitabine can inhibit the expression of CTLA-4 in CRC cells and might bridge the immunotherapy approaches with chemotherapy.

## 1. Introduction

CRC is responsible for the third cause of cancer-related death among men and the second cause of cancer-related death among women [1]. Additionally, approximately 50 percent of newly diagnosed CRC patients develop metastasis, which is associated with an inferior prognosis [2]. Therefore, there is an urgent need to further our knowledge of CRC biology to inhibit oncogenic signals in CRC.

Inhibitory immune checkpoints can inhibit the development of anti-tumoral immune responses and pave the way for tumor proliferation and metastasis. CTLA-4/CD80, CD86 axis, which can be established between immune cells and tumoral cells, has been implicated in attenuating the development of anti-tumoral immune responses in the tumor microenvironment. Indeed, the satisfactory results of CTLA-4 inhibitors in rejecting various tumors have translated into the FDA’s approval to treat patients with cancers [3]. Consistent with this, a recent meta-analysis has indicated that the gene expression of CTLA-4 can be associated with the inferior prognosis of affected patients (hazard ratio (HR): 1.50, 95% CI: 1.20–1.86) [4]. Therefore, targeting this pro-tumoral axis might be a promising strategy to improve the prognosis of CRC patients.

Chemotherapy has been one of the mainstream approaches for treating patients with cancers. As one of the effective chemotherapeutic agents for treating CRC, capecitabine is designed to inhibit the fast-paced tumor proliferation via interrupting the cell cycle and inducing apoptosis [5,6]. In addition to the cytotoxic effect of chemotherapeutic agents on the cell cycle and apoptosis of tumoral cells, they can substantially alter the expression of inhibitory immune checkpoint molecules in tumoral cells and regulate anti-tumoral immune responses. It has been reported that fluorouracil (5-FU) can upregulate programmed death-ligand 1 (PD-L1), as another inhibitory immune checkpoint molecule, in CRC cells [7]. However, the effect of capecitabine on the tumoral CTLA-4 in CRC cells has not been well-studied.

This study aimed to use clinical specimens and bioinformatic tools to assess the CTLA-4 expression in cancer and adjacent samples. Moreover, we intended to investigate the effect of capecitabine on CTLA-4 expression in the SW480 cells. The results of this study might further our knowledge of capecitabine on the tumoral CTLA-4 expression and might bridge the immunotherapy approaches with chemotherapy.

## 2. Materials and Methods

### 2.1. Evaluation of CTLA-4 Expression in CRC—In Silico Study

#### 2.1.1. CTLA-4 Expression in GEO Datasets

Data preprocessing is a data mining technique that involves transforming raw data into an understandable format. The GSE110224 and GSE25070 microarray datasets were downloaded from the Gene Expression Omnibus (GEO) database (https://www.ncbi.nlm.nih.gov/geo/ (accessed on 1 May 2021)). The GSE110224 dataset was based on GPL570 [HG-U133_Plus_2] Affymetrix Human Genome U133 Plus 2.0 Array with 34 samples (17 patients with CRC and 17 adjacent samples). The GSE25070 dataset, based on the Illumina HumanRef-8 v3.0 expression bead chip, included 26 CRC samples and 26 adjacent non-tumor colorectal tissue samples. The raw data were corrected and quantile-normalized with the affy package of R 3.4.1 in Bioconductor [8,9]. The annotation file published by Affymetrix was applied to assign probes to gene IDs and symbols. Data of probe IDs that could not be converted were excluded. Then, the average expression data of identifiers were obtained for each sample. The heatmap was plotted using the pheatmap R package for the CTLA-4 gene in two different CRC datasets.

#### 2.1.2. CTLA-4 Expression UCSC Xena Browser

For initial validation of CTLA-4 expression in primary tumors, normal solid tissue, recurrent tumor, and metastatic tumors, data were extracted from the TCGA-COAD database using the UCSC Cancer Browser (https://xenabrowser.net/ (accessed on 1 May 2021)) Then, CTLA-4 expression in HT-29, HCT 116, and SW480 cells was obtained from the Cancer Cell Line Encyclopedia (CCLE) datasets using the Xena browser. Finally, survival analysis of the CTLA-4 gene was demonstrated in Kaplan–Meier survival curves.

### 2.2. Evaluating CTLA-4 Expression in CRC—In Vitro Study

#### 2.2.1. Patients and Samples

This study was approved by the Ethics Committee of Tabriz University of Medical Sciences (IR.TBZMED.REC.1399.293). All participants signed written informed consent before the surgery. Eighteen samples of colorectal and adjacent non-tumoral samples were included in the study. Inclusion criteria for this study were individuals with age ≥ 30 years, without a history of inflammatory diseases, without a known hereditary predisposition to CRC, or other overwhelming disorders. All included CRC patients were histopathologically confirmed by experienced pathologists. The fresh samples were collected and transported in a nitrogen tank from the operation room to the laboratory. Diethyl pyruvate carbonate (DEPC) was used to remove RNase.

#### 2.2.2. Cell Culture

Three CRC cell lines, i.e., HT-29, HCT 116, and SW480, were purchased from the Pasture Institute of Iran. These cells were cultured in RPMI-1640 medium supplemented with 10% fetal bovine serum (FBS), penicillin, and streptomycin (Gibco, Carlsbad, CA, USA) in an incubator with 5% CO_2_ at 37 °C.

#### 2.2.3. CTLA-4 Expression in HT-29, HCT 116, and SW480 Cells

The HT-29, HCT 116, and SW480 cells (35 × 10^4^) were seeded in six-well plates and incubated at 37 °C in 5% CO_2_. After 24 h, the cells were harvested using trypsin/EDTA, centrifuged at 1500 g for 15 min, and prepared for RNA extraction, cDNA synthesis, and real-time PCR.

#### 2.2.4. Cell Proliferation Assays Using Capecitabine

The 15 × 10^3^ SW480 cells were cultured in each well at a confluency level of 75% for 24 h. Then, they were treated with different concentrations of capecitabine for 24 h. After 24 h, 3-(4,5-dimethylthiazol-2-yl)-2,5-diphenyl-2H-tetrazolium bromide (MTT) was added to the wells for 4 h (5 mg/mL). After removing the medium, 150 μL dimethyl sulfoxide (DMSO) was added to each well, and the plate was shaken for 10 min. A microplate reader (Tecan Sunrise, Männedorf, Switzerland) at a test wavelength of 570 nm and a reference wavelength of 630 nm was used to obtain the information.

#### 2.2.5. RNA Extraction and Complementary DNA (cDNA) Synthesis

According to the manufacturer’s protocol, the whole RNA from colorectal tissue and marginal samples was extracted using Trizol reagent (RiboEx). Before RNA extraction, whole tissues were placed in a tissue homogenizer on Trizol and squashed using a tissue homogenizer with Serrated-Pestle (Grinded Vessel) on ice several times. The cDNA synthesis kit (Biofact, Daejeon, South Korea) was used to synthesize cDNA.

#### 2.2.6. Real-Time PCR Analysis

A 2X Master Mix with high ROX (Biofact) was used for real-time PCR analysis. Three runs were conducted for each experiment. The PCR condition was as follows: initial denaturation for 13 min at 95 °C; 45 cycles of denaturation for 13 s, 95 °C; annealing, 30 s, 60 °C; elongation, 20 s, 72 °C. The specific primer sequences were used as follows: CTLA-4 (F: 5′-CATGATGGGGAATGAGTTGACC-3′, R: 5′-TCAGTCCTTGGATAGTGAGGTTC-3′) and GAPDH (F:5′-AAGGTGAAGGTCGGAGTCAAC-3′, R:5′-GGGGTCATTGATGGCAACAA-3′). In the current research, GAPDH was used as an internal control.

#### 2.2.7. Electrophoresis for Real-Time PCR Products

After real-time PCR analysis, electrophoresis was carried out to detect the CTLA-4 and GAPDH gene expressions in the SW480 cells before and after treatment with capecitabine; 3% agarose gel was used for this purpose.

### 2.3. Statistical Analysis

GraphPad Prism 6.0 (GraphPad Software, San Diego, CA, USA) was used to analyze the data. The paired t-test was used to compare the data with the controls, and one-way ANOVA was used to determine if there were statistically significant differences in the comparison of multiple groups. R version 3.4.1 was used for the in-silico analysis. *p* < 0.05 was considered a criterion for statistical significance between groups.

## 3. Results

### 3.1. Data Preprocessing and Heatmaps of CTLA-4 Expression in GSE110224 and GSE25070 Datasets

**Figure 1:**
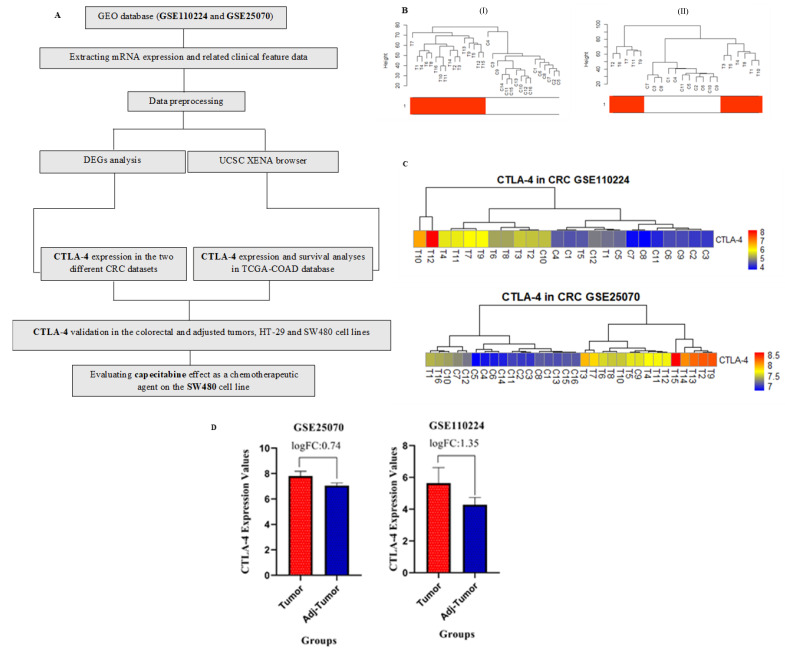
Data preprocessing, heatmaps of cytotoxic T-lymphocyte-associated protein 4 (CTLA-4) expression in GSE110224, and GSE25070 datasets and CTLA-4 expression in cancer cells and adjacent. (**A**) The flowchart of data preparation, processing, and analysis in this study. (**B**) Sample dendrogram and trait heatmap. (I) GSE25070 and (II) GSE110224 dataset; the color is proportional to the pathological stage (red = primary CRC and white = normal adjacent sample). (**C**) The expression analysis of CTLA-4 in different groups has shown that this gene is highly expressed in colorectal cancer (CRC) cells compared to the adjacent cells. (**D**) CTLA-4 expression in both datasets has been higher in the cancer samples than in the controls, C: control, and T: tumor.

We performed quantile normalization to reduce the effects of technical noises. Probe ID conversion and averaging probes were conducted as mentioned in the previous study [10]. Figure 1A aims to demonstrate the process of data analysis. After hierarchical clustering, six patients were outliers to GSE110224, and ten patients were defined as outliers for the GSE25070 dataset (Figure 1B).

CTLA-4 expression was evaluated in two datasets, i.e., GSE110224 and GSE25070. We used heatmap analysis to describe the values of CTLA-4 in the samples of these two datasets. The results demonstrated significant expression patterns in the samples (Figure 1C). As shown in Figure 1D, CTLA-4 expression in both datasets was significantly higher in the cancer samples than in the control ones. The logFC was 0.74 (adjusted *p*-value = 1.91 × 10^−6^) for GSE25070 and 1.35 (adjusted *p*-value = 0.00095) for GSE110224 dataset.

### 3.2. CTLA-4 Expression and Kaplan–Meier Survival Analysis Using UCSC Xena Browser

**Figure 2:**
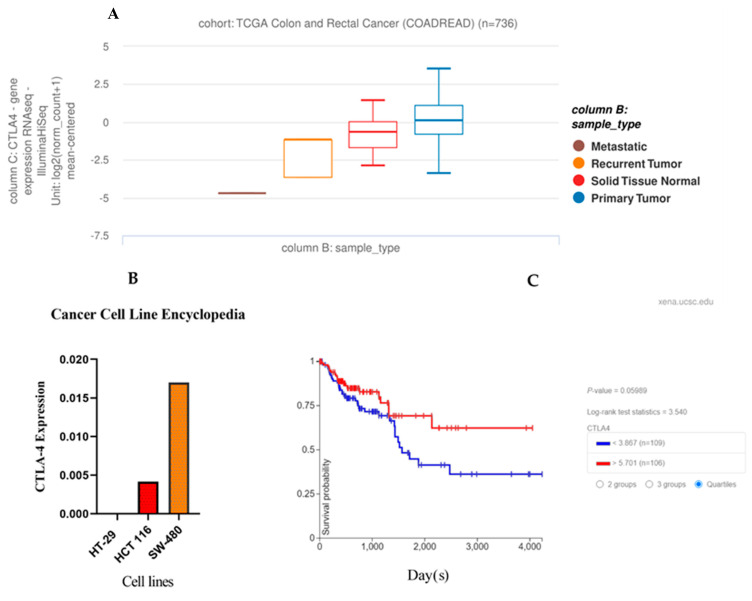
Obtained data from the cancer genome atlas (TCGA) and cancer cell line encyclopedia (CCLE). (**A**) Cytotoxic T-lymphocyte-associated protein 4 (CTLA-4) expression profiles and clinicopathological data of patients with CRC. (**B**) The CCLE showed a high CTLA-4 expression level in SW480 cells. (**C**) Overall survival of colorectal cancer (CRC) patients stratified by CTLA-4 expression levels (the cut-off criterion is lower than 3.867 and higher than 5.701).

We used RNA-sequencing data for the initial analysis of the CTLA-4 gene expression in CRC and non-tumoral samples. The clinicopathological and survival data of patients with primary CRC were obtained from the UCSC Xena browser (https://xenabrowser.net/ (accessed on 1 May 2021)). As shown in Figure 2A, CTLA-4 expression was significantly different in primary CRC, non-tumoral samples, recurrent tumors, and metastatic tumors (N = 736; metastatic = 1 sample, recurrent tumor = 2 samples, primary tumor = 632 samples, and solid tissue normal = 101 samples, *p* = 2.1 × 10^−7^). Our results showed that CTLA-4 gene expression was upregulated in the primary CRC compared to non-tumoral samples.

Based on the bioinformatics analysis, CTLA-4 expression was substantially higher in the SW480 cells than in the HT-29 and HCT 116 cells (Figure 2B). After considering the cut-off point, the Kaplan–Meier survival analysis was performed based on CTLA-4 expression. Our results failed to show any significant prognostic value for CTLA-4 expression in determining the overall survival of CRC patients (*p* = 0.059) (Figure 2C).

### 3.3. Clinicopathological Features of Included CRC Patients

Eighteen patients with CRC were enrolled in the current study. Table 1 demonstrates the clinicopathological features of the included patients.

### 3.4. The CTLA-4 Expression in CRC Tissues and Adjacent Non-Tumoral Samples

**Figure 3:**
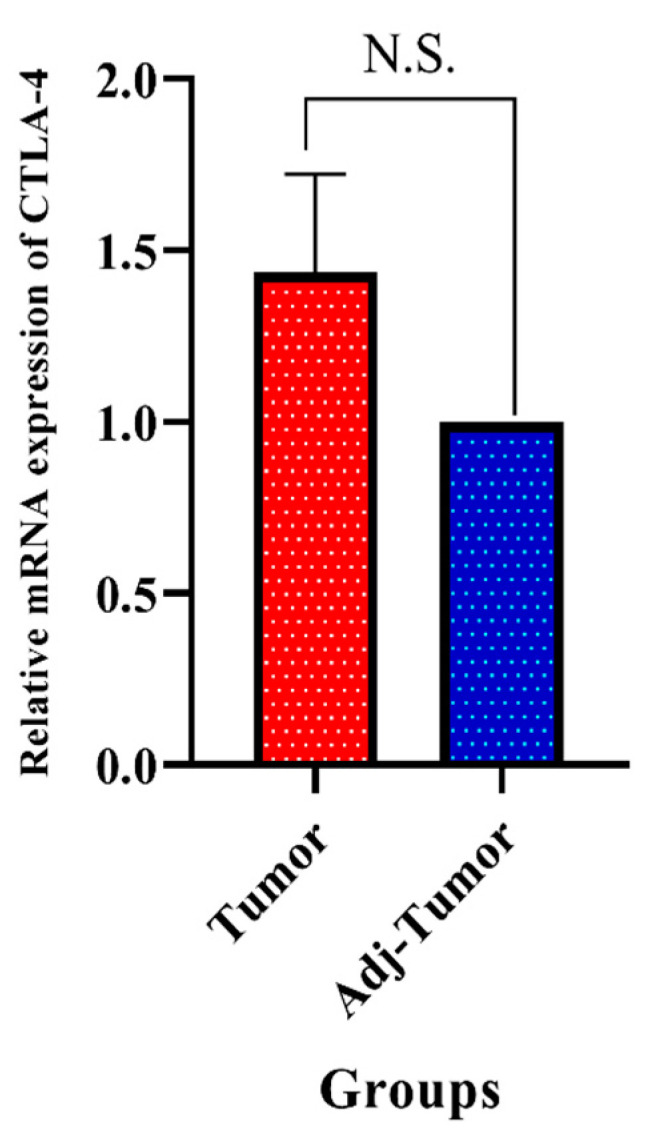
Cytotoxic T-lymphocyte-associated protein 4 (CTLA-4) expression was higher in tumor tissues than in the adjacent non-tumoral tissues; however, this change was not statistically significant; N.S: non-significant, Adj: adjacent.

Real-time PCR was used to analyze the relative expression of CTLA-4 in CRC and adjacent non-tumoral tissues via the 2^ ^(−ΔΔ CT)^ method. Although our results showed a trend in the increased expression of *CTLA-4* in the CRC tissues, this change was not statistically significant (*p* = 0.202) (Figure 3).

### 3.5. The Comparison of CTLA-4 Expression in HT-29 and SW480 Cell Lines

**Figure 4:**
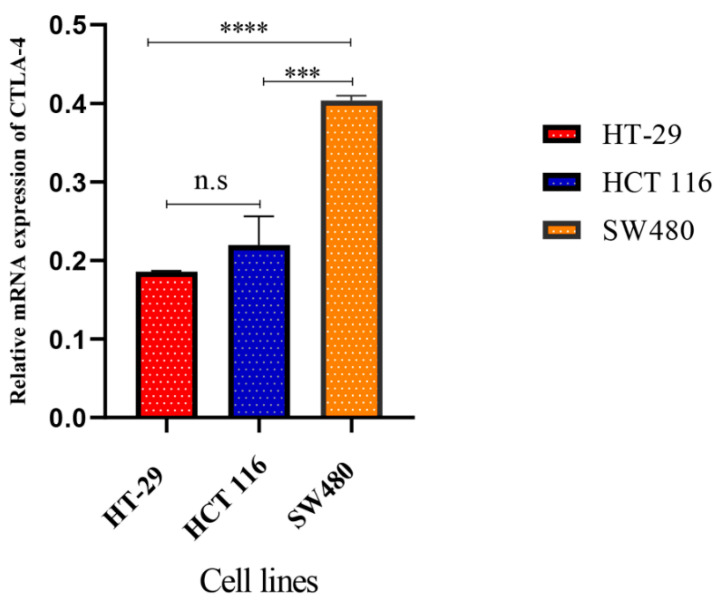
The gene expression of cytotoxic T-lymphocyte-associated protein 4 (CTLA-4) in HT-29, HCT 116, and SW480 cells. The real-time quantitative reverse transcription PCR (qRT-PCR) assay has indicated that CTLA-4 expression in the SW480 cells is substantially higher than the HT-29 and HCT 116 cells. N.S: non-significant, *** *p* < 0.001, and **** *p* < 0.0001.

The real-time PCR was used to measure the CTLA-4 expression in CRC cell lines, including the HT-29, HCT 116, and SW480 cell lines. Our results showed that the CTLA-4 was significantly overexpressed in SW480 cells compared to the HT-29 and HCT 166 cells (*p* < 0.0001 and *p* < 0.001, respectively) (Figure 4).

### 3.6. The Effect of Capecitabine on the Viability of SW480 Cells

**Figure 5:**
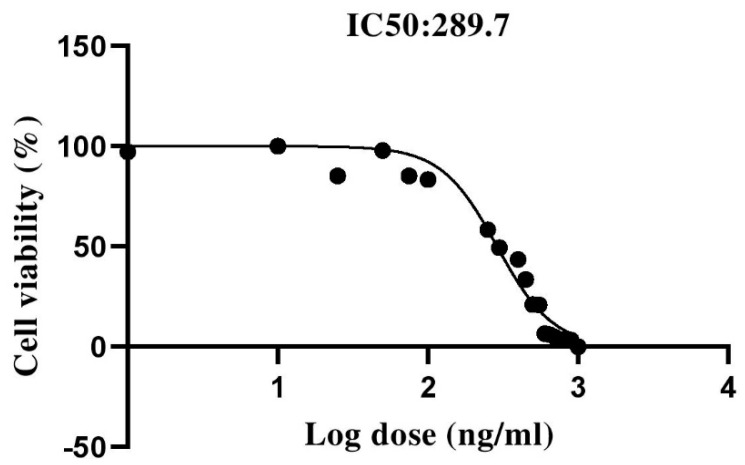
The dose–response and cell viability curve of SW480 cells. The results indicated that the half-maximal inhibitory concentration (IC50) of capecitabine in the SW480 cells was 289.7 ng/mL.

Our results showed that capecitabine could decrease the viability of SW480 cells in a dose-dependent manner. The MTT results indicated that 289.7 ng/mL was the half-maximal inhibitory concentration (IC50) of capecitabine for SW480 cells. This value was used for conducting experiments to determine the effect of capecitabine on the CTLA-4 gene expression in the SW480 cells (Figure 5).

### 3.7. CTLA-4 Expression in SW480 Cells after Treatment with Capecitabine

**Figure 6:**
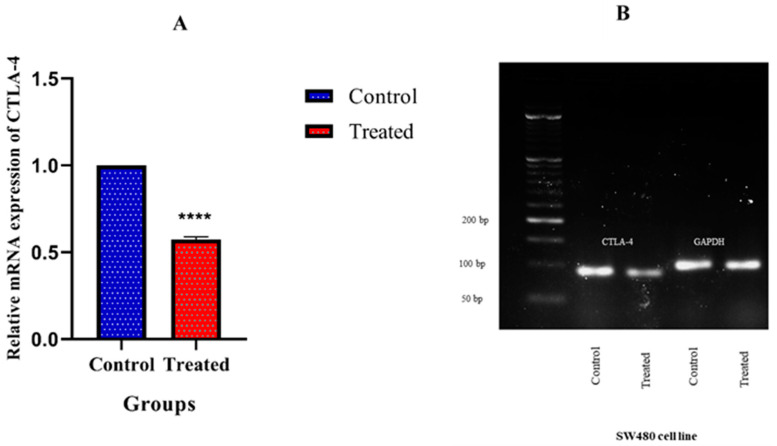
The mRNA expression of cytotoxic T-lymphocyte-associated protein 4 (CTLA-4) in the SW480 cells. (**A**): The CTLA-4 expression has been significantly lower in the control group compared to the capecitabine-treated group. Error bars display the standard deviation. (**B**): Gel electrophoresis of CTLA-4 (92 bp) and GAPDH (102 bp). From right to left, lines show DNA ladder (50–1500 bp), CTLA-4 expression in the SW480 cell line (control), CTLA-4 expression after treatment with capecitabine. The other lanes are related to glyceraldehyde-3-phosphate dehydrogenase (GAPDH) (control and treated, respectively); **** *p*-value < 0.0001.

The real-time PCR was used to measure the mRNA expression of CTLA-4 in the control and capecitabine-treated group. Our results demonstrated that capecitabine could significantly decrease CTLA-4 expression (*p* < 0.0001) (Figure 6A). The products of the real-time PCR were detected using electrophoresis (Figure 6B).

## 4. Discussion

Despite remarkable advances in understating CRC biology and their interaction with the tumor microenvironment, the current approaches have not brought a treatment with the highest response rate and the lowest side effects for affected patients. A better understanding of the interaction between tumor microenvironment with CRC cells might pave the way for developing a more efficient therapy for CRC patients [11]. Our bioinformatic results have shown that CRC cells can substantially overexpress CTLA-4 compared to the adjacent non-tumoral cells. Our in vitro studies have shown that capecitabine can remarkably decrease the gene expression of CTLA-4 in SW480 cells, which might bridge chemotherapy and immunotherapy in CRC.

Initial studies have shown that CTLA-4 can be expressed in tumor-infiltrating lymphocytes, leading to the attenuation of anti-tumoral immune responses. However, recent findings have demonstrated that CTLA-4 can also be expressed on tumoral cells and shield tumor cells from immune responses [12,13]. Liu et al. have shown that CTLA-4 overexpression is associated with higher tumor grade and worse overall survival of glioma patients [12]. Indeed, the CTLA-4 expression has been demonstrated on other slid cancers, such as non-small cell lung cancer, melanoma, and CRC. However, inhibiting tumoral CTLA-4 has resulted in PD-L1 upregulation and tumor relapse [13,14]. Omura et al. have shown that the overexpression of membrane-bound CTLA-4 and membrane-bound PD-L1 in CRC cells can be associated with worse overall survival and disease-free survival (HR = 3.86, 95%, CI: 1.71–8.51, *p*-value = 0.001, and HR = 2.53, 95%, CI: 1.23–4.95, *p*-value = 0.01, respectively) [15]. Although our bioinformatic results have shown that CTLA-4 can be overexpressed in CRC tissues compared to the non-tumoral adjacent tissues, our clinical samples have failed to show any statistically significant differences between CRC tissues and adjacent non-tumoral tissues, which might be stemmed from our low sample size (*p*-value > 0.05). Our bioinformatic results have failed to depict a statistically significant figure for the prognostic value of CTLA-4 for determining the overall survival of CRC patients (*p*-value = 0.059).

Although identifying immune checkpoint axes have opened up a new era for cancer therapy, monotherapy with immune checkpoint inhibitors has not shown the efficacy as would be expected. The disparity between response rates of immunotherapy approaches between patients has not been ignorable. Although the advances in the single-cell sequencing techniques might bring novel solutions for improving the response rates of immunotherapy approaches, one of the currently accepted approaches is the combination of chemotherapy approaches with immune checkpoint inhibitors [16,17]. Therefore, a better understanding of chemotherapeutic agents on the tumor microenvironment might be key for its clinical translation. Capecitabine is a 5-FU related oral product that primarily targets thymidylate synthase resulting in the disruption of DNA synthesis in rapidly proliferating cells [18]. Javadrashid et al. have shown that 5-FU treatment can downregulate the expression of tumoral PD-L1 in pancreatic cancer cells [19]. However, Van Der Kraak et al. have demonstrated that 5-FU treatment can induce the overexpression of PD-L1 in CRC cells [7]. Our results have indicated that capecitabine, as a prodrug of 5-FU, can substantially inhibit the tumoral CTLA-4 in the SW480 cells.

Our study has several strengths. First, our study has used bioinformatics, clinical samples, and cellular studies to address the research question. Second, the current study aimed to provide a blueprint for further investigations regarding the combination value of immune checkpoint inhibitors and capecitabine. However, the present study has several limitations as well. First, our small sample size and lack of an organized registration system did not allow us to include more CRC patients and provide survival analysis. Second, we could not measure the protein expression of CTLA-4 following treating the SW480 cells with capecitabine.

## 5. Conclusions

Based on GSE110224 and GSE25070 datasets, we have found that CTLA-4 expression is substantially upregulated in CRC tissues compared to adjacent non-tumor samples. Although the expression of CTLA-4 has been remarkably different among primary CRC, non-tumoral samples, recurrent tumors, and metastatic tumors. Our results have failed to demonstrate a significant prognostic value for CTLA-4 expression for determining the overall survival of CRC patients. Based on our eighteen CRC samples, we have found a trend in the increased expression of CTLA-4 in CRC tissues compared to the adjacent non-tumoral tissues; however, this change was not statistically significant. We have found that SW480 cells overexpress CTLA-4 compared to HT-29 and HCT 116 cells, and treating SW480 cells with the IC50 of capecitabine, 289.7 ng/mL, can considerably decrease CTLA-4 expression in SW480 cells. Further in vivo analysis and animal model studies are needed to reveal the exact effect of capecitabine on the CTLA-4 expression in CRC patients.

## Figures and Tables

**Table 1:** Characteristics of the study population.

Clinicopathological Characteristics	N (%)
Female, sex	8 (44.44%)
Age years, mean (range)	59 (30–79%)
Location, colon	12 (66.6%)
Location, rectum	6 (33.4%)
liver metastasis	5 (27.7%)
Stage I	4 (22.2%)
Stage II	1 (5.6%)
Stage III	9 (50%)
Stage IV	4 (22.2%)

## Data Availability

Not available.
